# Clinical Decision Support Systems Using Home Blood Pressure Readings to Manage Patients With Hypertension: Scoping Review

**DOI:** 10.2196/75551

**Published:** 2025-10-03

**Authors:** Aminath Shiwaza Moosa, Jun Jie Benjamin Seng, Chirk Jenn Ng

**Affiliations:** 1 SingHealth Polyclinics Singapore Singapore; 2 Family Medicine Academic Clinical Programme SingHealth Duke-NUS Academic Medical Centre Singapore Singapore; 3 Ministry of Health Holding (Singapore) Singapore Singapore; 4 SingHealth Centre for Population Health Research and Implementation Singapore Singapore

**Keywords:** clinical decision support system, hypertension, scoping review, home blood pressure, self-monitored blood pressure, telemonitoring, decision support

## Abstract

**Background:**

Home blood pressure (HBP) is an important parameter that guides clinicians in managing hypertension in patients. However, in using these records to manage patients, physicians face challenges, particularly regarding access, integration, and interpretation of the records when making clinical decisions. Clinical decision support systems (CDSSs) have been proposed to address these challenges; however, current literature reveals significant heterogeneity and gaps in CDSSs used for hypertension management.

**Objective:**

This study aimed to summarize existing studies on CDSSs that use HBP readings to manage patients with hypertension.

**Methods:**

We conducted a scoping review, with searches performed in PubMed, Embase, and Scopus on April 1, 2024. The results were reported in accordance with the PRISMA-ScR (Preferred Reporting Items for Systematic Reviews and Meta-Analyses extension for Scoping Reviews) checklist. Studies that used CDSSs integrated with HBP monitoring among adult patients with hypertension in outpatient settings were included. Non-English studies were excluded. Outcomes assessed included the theoretical frameworks used for CDSS development, CDSS components (data capture, processing, and output), clinical outcomes, user experiences, and implementation processes.

**Results:**

Of the 5023 articles screened, 33 (0.66%) were included. Most of the studies were conducted in the United States (16/33, 49%) and were randomized controlled trials (21/33, 64%). Nearly two-thirds of the CDSSs (21/33, 64%) were computerized. Only 1 (3%) of the 33 studies reported using a theoretical framework for CDSS development. HBP recording and uploading were predominantly automatic (23/33, 70%). All computerized CDSSs (21/33, 64%) used rule-based algorithms, and most (19/21, 91%) incorporated alert triggers for results outside the reference range. More than a third of the studies (13/33, 39%) were based on hypertension guidelines. Among studies that reported outcomes, most reported improved blood pressure (25/29, 86%) and adjustment in antihypertensive medications (16/19, 84%). Patients and clinicians appreciated the convenience and remote monitoring (10/33, 30%) but reported challenges with usability and access to computerized CDSSs (2/21, 10%). Of the studies using noncomputerized CDSSs (12/33, 36%), all incorporated patient education, while nearly two-thirds of the studies using computerized CDSSs (13/21, 62%) did the same. Clinician training was reported in 5% (1/21) of the computerized CDSSs and 25% (3/12) of the noncomputerized CDSSs.

**Conclusions:**

While CDSSs hold promise for improving hypertension management, gaps remain in their development and implementation. Future efforts should focus on integrating robust frameworks; aligning with guidelines; enhancing manual data integration; and addressing usability to maximize effectiveness, adoption, and user satisfaction.

**Trial Registration:**

Open Science Framework 26zmn; https://osf.io/26zmn

## Introduction

### Background

Self-monitoring of diseases with support from patients’ health care providers is crucial in managing many chronic conditions and their risk factors, such as hypertension [1-3]. International guidelines on hypertension recommend self-monitoring of home blood pressure (HBP) for diagnosing and monitoring of hypertension because self-monitoring is widely accessible, cost-effective, and easy to use [4]. HBP monitoring offers the advantage of detecting white coat hypertension (falsely elevated office BP) and masked hypertension (falsely normal office BP), phenotypes that office BP measurements miss [5]. HBP has also been shown to be a predictor of cardiovascular outcomes independent of office BP measurements [6,7].

Despite the recommendations, clinicians face significant challenges in managing patients with hypertension due to difficulties in interpreting and documenting HBP records, the absence of local guidelines, and a lack of training in managing BP variability [8]. In clinical practice, HBP records are available to clinicians in different forms, including handwritten paper diaries, smartphone-captured logs, and dashboard records. Examining and manually analyzing these HBP records and comparing them with office BP can be time consuming in busy primary care clinics, which leads to inaccuracies in BP assessment and management [8]. A recent qualitative study exploring barriers to hypertension management in primary care found significant variations in clinicians’ interpretation of HBP records, which can lead to erroneous decision-making in the diagnosis and treatment of hypertension [9]. In addition, HBP monitoring results in improved BP control when combined with cointerventions such as timely adjustments in antihypertensive medications by clinicians or guided self-titration by patients, patient education, or lifestyle counseling [10].

Clinical decision support systems (CDSSs) have been used to support clinical decision-making, including in hypertension management [11-14]. A CDSS is a specialized decision support system designed to assist health care professionals in making informed decisions about patient care [15]. It aims to reduce variation in health care delivery, automate time-consuming and cognitively demanding tasks, reduce challenges caused by competing priorities (such as patient concerns vs health maintenance), and bridge gaps in clinician knowledge of guidelines [12]. In the context of hypertension, a CDSS can detect elevated BP readings at clinic visits and support clinicians in diagnosing hypertension, providing lifestyle counseling, and adjusting medication; this is particularly helpful in fast-paced primary care clinics. In addition, a CDSS can help clinicians interpret HBP data by providing average HBP values for making informed decisions about treatment and lifestyle changes.

Current literature reveals significant heterogeneity and gaps in CDSSs used for hypertension management [12]; considerable variation exists in CDSS types and functionality (eg, ranging from simple alerts to complex telehealth platforms) [13,14], development processes (eg, different theoretical frameworks used), outcome measures (eg, BP control and medication adjustment), and implementation (eg, training and workflow integration). A comprehensive literature review is crucial to address these challenges and inform the development of a robust and effective CDSS for HBP-supported hypertension management.

### Objectives

This scoping review aimed to address these gaps by mapping existing CDSSs for hypertension and examining their features, development processes, outcome measures, and implementation. This will inform the development of a CDSS that addresses potential challenges while leveraging current enablers.

## Methods

### Overview

We conducted a scoping review for studies that evaluated the use of CDSSs for managing patients with hypertension using HBP readings. We reported this review in accordance with the PRISMA-ScR (Preferred Reporting Items for Systematic Reviews and Meta-Analyses extension for Scoping Reviews) checklist [16]. The protocol for this scoping review has been registered in Open Science Framework (26zmn) [17].

### CDSS Types

A CDSS can be computerized or noncomputerized. A computerized CDSS uses algorithms or machine learning to process data; these include electronic health record systems with built-in decision support or artificial intelligence–driven diagnostic tools. A noncomputerized CDSS requires manual processes where human intervention is necessary to analyze and interpret data; these include clinical guidelines, traditional flowcharts, or checklists used by health care providers [18].

**Figure 1:**
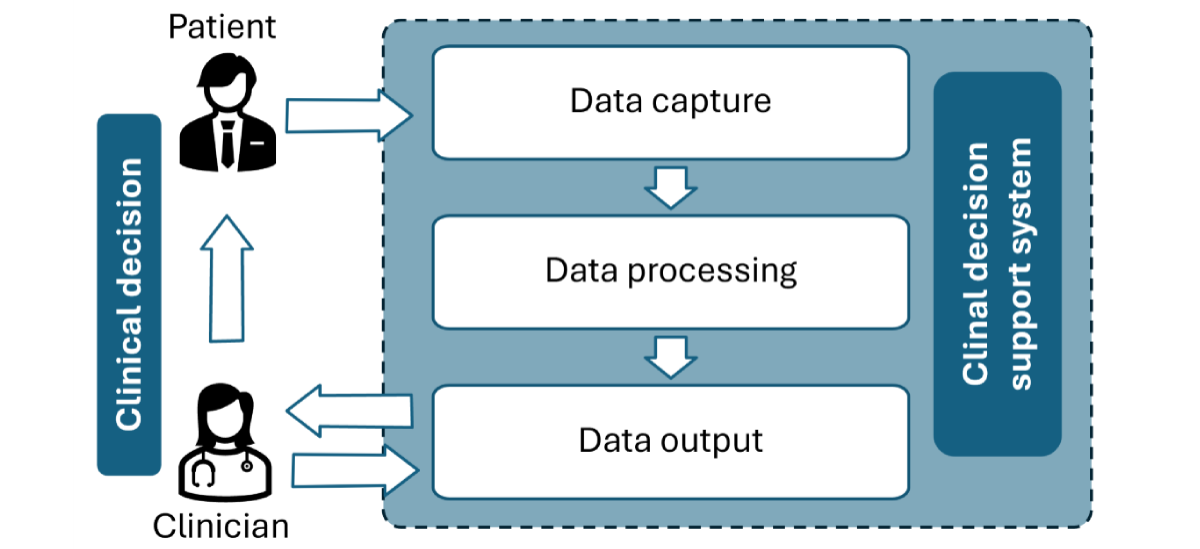
Key components of the clinical decision support system and their interactions.

A CDSS consists of 3 components: data capture, processing, and output. These components are typically associated with computerized CDSSs; however, in this review, they are applied to both computerized and noncomputerized CDSSs (Figure 1).

Data capture involves gathering accurate, complete, and timely patient-related information from internal and external sources [19]. Ensuring high-quality and relevant data before input into the CDSS is crucial, as a reliable data acquisition process provides the CDSS with up-to-date information to generate appropriate and safe recommendations [19].

Data processing encompasses models that process and analyze the captured data. The selection of appropriate models is essential to ensure the accuracy and effectiveness of the CDSS. These models must be clinically validated and tailored to specific decision support needs, such as diagnosis, treatment, or risk assessment. CDSSs use various models to support clinical decision-making, which can be broadly classified into knowledge-based [20] and nonknowledge-based systems [21]. Knowledge-based systems use clinical practice guidelines, evidence-based medicine, or expert opinion as their knowledge base. This knowledge base is encoded as rules (if-then statements) or decision trees, which are applied to patient data to trigger alerts or generate recommendations [20]. By contrast, nonknowledge-based systems leverage artificial intelligence or statistical patterns instead of rule-based algorithms; they use predictive models or pattern recognition to interpret data in a clinically meaningful way [21].

On the basis of the analysis, the data output provides actionable advice and evidence-based recommendations through a user interface to support health care providers in making informed decisions regarding risk assessment, diagnosis, and treatment. To increase uptake and sustainability, the CDSS user interface must be user-friendly, have features that explain the basis for the recommendations, and provide a platform for clinicians’ feedback.

### Information Sources and Search Strategy

We searched PubMed, Scopus, and Embase on April 1, 2024. In addition, relevant gray literature was exploredthrough expert consultation, and citations from the included articles were screened to capture potentially relevant studies missed in the initial search.

The search strategy encompassed Medical Subject Headings (MeSH) and search terms related to “hypertension,” “clinical decision support systems,” and “home blood pressure monitoring.” As Scopus does not support controlled vocabulary indexing, the search was conducted using terms from the title, abstract, and keyword fields. The Embase search was restricted to the Embase database to avoid citations being retrieved from MEDLINE and PubMed. Search terms were adapted from other relevant scoping reviews [12,22]. The complete database search strings are provided in Multimedia Appendix 1.

The search period spanned from the inception of each database to April 1, 2024.

### Eligibility Criteria

We included full-text articles in English that evaluated the use of CDSSs for managing hypertension using self-monitored HBP readings. For this review, a CDSS was defined as any tool or intervention designed to enhance health care delivery by augmenting medical decisions with relevant clinical knowledge, patient information, and other health data [23]. All types of CDSSs were eligible, including computerized and noncomputerized CDSSs as well as knowledge-based and nonknowledge-based systems. The review focused on adult patients with hypertension aged 18 years or older. Eligible study designs included randomized controlled trials, observational studies, cross-sectional studies, cohort studies, and qualitative studies.

We excluded articles in which CDSSs did not incorporate self-monitored HBP readings into clinical decision-making, articles that focused solely on CDSS development rather than evaluation, editorials, case series, letters, and irrelevant systematic reviews and meta-analyses (Table 1).

**Table 1:** Eligibility criteria for studies included in the review.

Variables	Inclusion criteria	Exclusion criteria
Population	Adult (aged ≥18 y) patients with hypertension	Children, pregnant women, and patients without hypertension
Intervention	Use of a CDSS^a^ (computerized or noncomputerized) for hypertension management using self-monitored home BP^b^ readings for decision-making	Did not use a CDSS or self-monitored home BP readings for decision-making in hypertension management; only reported development of the CDSS
Comparison	Usual care^c^	N/A^d^
Outcome	All outcomes	N/A
Study design	Randomized controlled trials, observational studies, cross-sectional studies, cohort studies, and qualitative studies	Editorials, case series, letters, and irrelevant systematic reviews and meta-analyses
Language	English	Other

^a^CDSS: clinical decision support system.

^b^BP: blood pressure.

^c^Studies that used a comparator group.

^d^N/A: not applicable.

### Selection of Sources of Evidence, Data-Charting Process, and Data Items

Citations retrieved from all databases were exported to Covidence (Veritas Health Innovation Ltd) [24], a web-based software platform for performing scoping and systematic reviews. Duplicate citations were removed using the in-application function. Two independent reviewers (ASM and JJBS) conducted the initial pilot screening of 200 records to assess consistency and align the screening process. The initial agreement rate was an acceptable 90%. Subsequently, all titles and abstracts were screened independently by the same reviewers. Disagreements were resolved through discussion, and when consensus could not be reached, a third reviewer (CJN) was involved for arbitration.

The two independent reviewers extracted relevant data from the included articles using Covidence, including study design, year of publication, participant characteristics, types and details of CDSSs, theoretical frameworks used for CDSS development, and outcome types.

Study authors were contacted to request missing data. If no response was received after two attempts, the data were recorded as missing. As this was a scoping review, studies with substantial missing data were not excluded to capture the full breadth of the literature.

### Critical Appraisal of Individual Sources of Evidence

A risk-of-bias assessment was not performed, as this scoping review aimed to capture and map the full spectrum of available literature on CDSSs.

### Summary and Synthesis of Results

Characteristics of the included studies were summarized using descriptive statistics.

Emphasis was placed on summarizing details related to the type of CDSS (computerized or noncomputerized); documentation, upload, and processing of HBP records; theoretical frameworks used for CDSS development; data processing and output; outcomes measured; and user experiences. CDSSs were classified as computerized if processing of HBP records required computer software and noncomputerized if HBP data were processed manually [18].

To standardize the reporting of CDSSs across all articles, the research team developed a 16-item checklist (“CDSS-hypertension”) with inputs from hypertension experts. The checklist covers the title, abstract, introduction (background and aim), methodology (study design, setting, participants, design and function of the CDSS, outcomes, and statistics), results, discussion (key results, interpretation, and limitations), and funding (Multimedia Appendix 2).

## Results

### Summary

**Figure 2:**
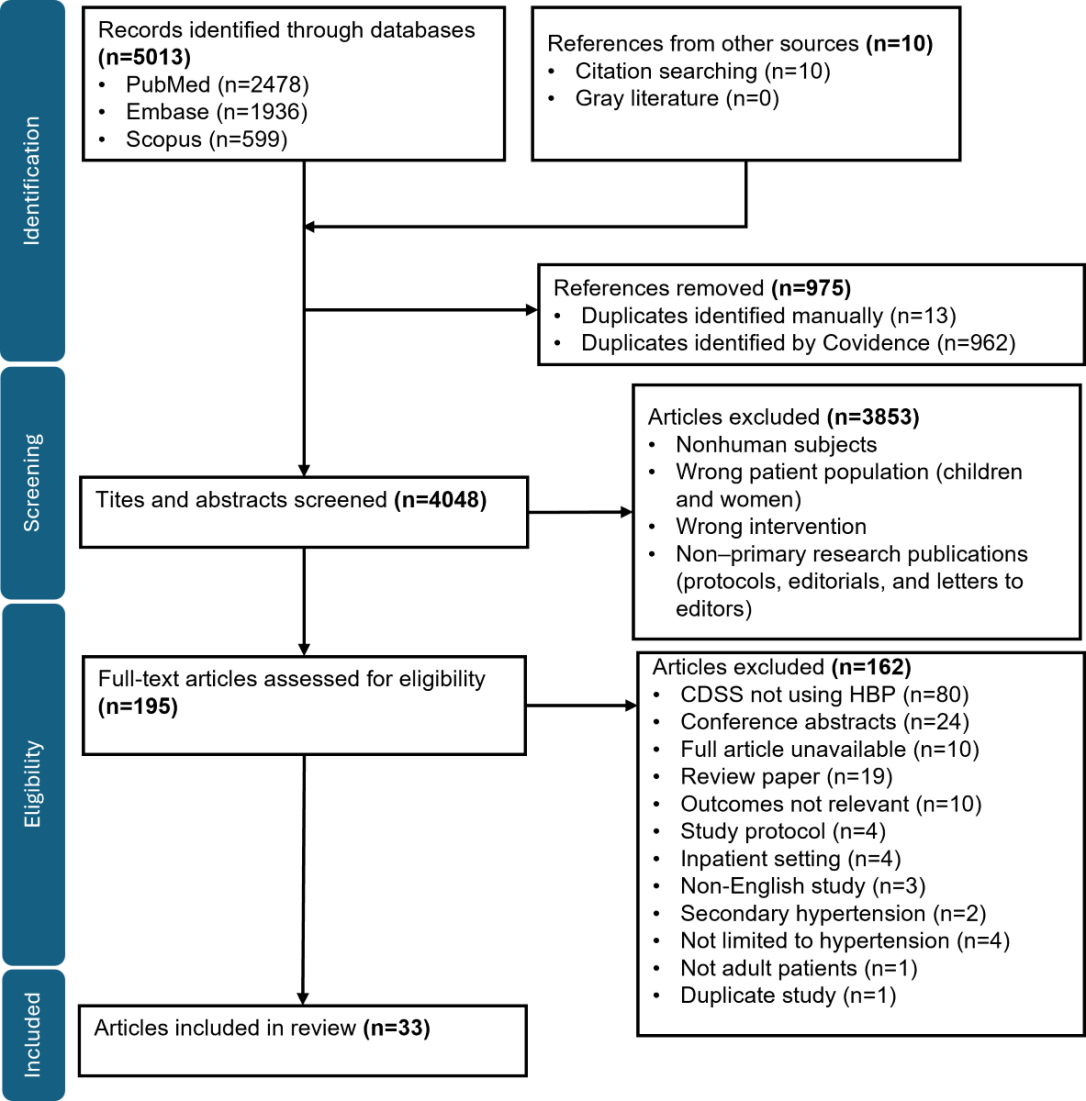
PRISMA (Preferred Reporting Items for Systematic Reviews and Meta-Analyses) flow diagram.

The literature search identified 5023 records, of which 33 (0.66%) full-text articles that reported results from 29 unique studies were included in this review (Figure 2).

### Study Characteristics

**Figure 3:**
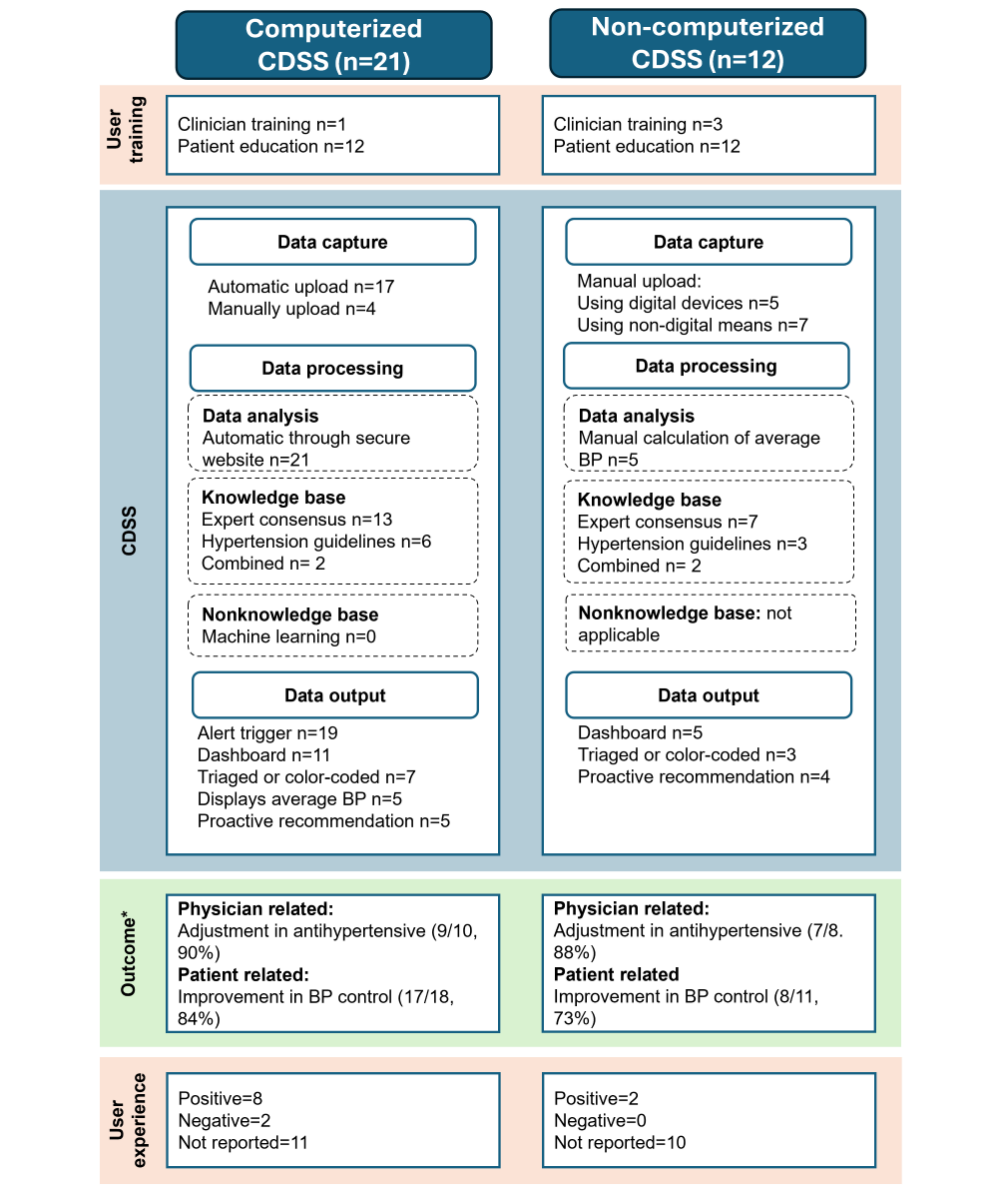
Summary of the scoping review results. BP: blood pressure; CDSS: clinical decision support system. *Outcome influenced by a combination of interventions and not exclusive to CDSS.

Table 2 depicts the characteristics of the included studies. The studies were conducted between 2000 and 2023. Most of the studies (16/33, 48%) [13,23,25-38] were conducted in the United States. Randomized controlled trials (21/33, 64%) [23,26-28,31-36,38-48] and cohort studies (5/33,15%) [13,30,49-51] were the most common study designs. Most of the studies (22/33, 67%) [13,14,25-27,29,31,32,34-37,39-44,46,47,51-53] were conducted in primary care or general practice. A summary of the scoping review results is shown in Figure 3, and detailed characteristics of the included studies are presented in Table S1 in Multimedia Appendix 3 [13,14,23,25-54].

**Table 2:** Characteristics of the included studies (n=33).

Characteristics			Studies, n (%)
**Country^a^**			
	North America (United States and Canada)		18 (54)
	Europe (Germany, Italy, Sweden, and United Kingdom^b^)		7 (21)
	Asia (China, Japan, Singapore, and Taiwan)		6 (18)
	South America (Brazil)		1 (3)
	Not reported		1 (3)
**Study design**			
	Randomized controlled trial		21 (64)
	Cohort study		5 (15)
	Quasi-experimental study		2 (6)
	Qualitative research		2 (6)
	Mixed methods study		1 (3)
	Quality improvement study		1 (3)
	Cross-sectional study		1 (3)
**Study setting**			
	Primary care and general practice		22 (67)
	Hospital, specialist outpatient clinic, or multispecialty clinic		7 (21)
	Not specified		4 (12)
**Theoretical frameworks**			
	Person-based approach		1 (3)
**Type of Home BP^c^** **recording and uploading**			
	Automatic		23 (70)
	Manual		10 (30)
**Source of the CDSS^d^ algorithm**			
	Expert consensus		20 (61)
	Hypertension guidelines		9 (27)
	Combined hypertension guidelines and expert consensus		4 (12)
**Type of CDSS**			
	Computerized		21 (64)
	Noncomputerized		12 (32)
**Outcomes^e^**			
	**Improvement in BP control^f^**		
		Yes	25 (86)
		No	4 (13)
	**Adjustments in antihypertensive medications^g^**		
		Yes	16 (89)
		No	2 (11)

^a^The United States contributed the greatest number of studies (16), followed by the United Kingdom (3), Canada (2), Japan (2), Singapore (2), Sweden (2), Germany (1), Italy (1), China (1), Taiwan (1), and Brazil (1). One study did not specify the country in which it was conducted.

^b^One study was conducted in Scotland.

^c^BP: blood pressure.

^d^CDSS: clinical decision support system.

^e^Outcomes influenced by a combination of interventions and not exclusive to CDSS.

^f^n=29.

^g^n=18.

### CDSS Development Process

Only 1 (3%) of the 33 included studies used a theoretical framework—a person-based approach—for CDSS development [42].

### CDSS Features

#### Type of CDSS

Almost two-thirds of the studies (21/33, 64%) used computerized CDSSs [13,14,26,29,31-35,37,39,40,42, 45-47,49-52,54], and one-third (12/33, 36%) used noncomputerized CDSSs [23,25,27,28,30,36,38,41,43,44,48].

#### Documenting and Uploading

BP recordings were predominantly automatic (23/33, 70%) [13,14,25-27,29-35,39-41,44-46,49-52,54]. Of the 21 computerized CDSSs, 4 (19%) required users to manually enter and upload their HBP records from a digital device to a dashboard or secure website [37,42,47,54], while 17 (81%) [13,14,26,29,31-35,39,40,45,46,49-52] automatically recorded and uploaded HBP records to a dashboard or secure website via BP devices using telephone lines, Bluetooth-enabled mobile apps, or web portals [13,14,26,29,31-35,39,40,45,46,49-52].

Half of the noncomputerized CDSSs (6/12, 50%) required patients to record their self-monitored BP values in paper diaries and submit them to physicians through various channels, including in-person visits [48], telephone [23,36], mail or secure SMS text messages [43], or by manually entering the data into a web portal [28,38]. The remaining half (6/12, 50%) involved transmitting HBP records directly from BP devices to the research team or health care provider via mobile devices or web portals using the internet [25,27,30,41,44,53].

#### HBP Processing

All studies on computerized CDSSs (21/33, 64%) processed HBP records through a secure website and used knowledge-based (rule-based) algorithms.

Of the 12 noncomputerized CDSSs, 5 (42%) required health care professionals or researchers to manually calculate the mean of the HBP records [25,36,41,44,53]. The remaining studies (7/12, 58%) did not indicate whether the mean BP was calculated [23,27,28,30,38,43,48].

#### Content (Sources)

Most of the CDSS algorithms (20/33, 61%) [14,25,27,29-36,38,44-48,50,52,54] were based on study-defined algorithms or expert opinion (Tables S2 and S3 in Multimedia Appendix 3). More than a quarter of the studies (9/33, 27%) were based on hypertension guidelines, including the Chinese national hypertension guidelines [53]; Seventh Joint National Committee on Prevention, Detection, Evaluation, and Treatment of High Blood Pressure guidelines [13,23,28,39,49]; European Society of Hypertension guidelines [51]; National Institute for Health and Care Excellence guidelines [41-43]; American Heart Association guidelines [26,37]; International Society of Hypertension guidelines [39]; and the Japanese Society of Hypertension guidelines and Hypertension Cardiovascular Outcome Prevention and Evidence in Asia Network recommendations [40]. In 4 (12%) of the 33 studies, the CDSSs were based on both hypertension guidelines and expert consensus [28,37,43,49].

#### Data Output

Most of the computerized CDSSs (19/21, 90%) incorporated an alert trigger for abnormal results [26,29,31-35,37,39,42,45-47,49-52,54]. Nearly half (11/21, 52%) featured a dashboard for health care providers to visualize the data [13,29,32-34,39,40,42,45,46,52]. One-third of the studies (7/21, 33%) triaged the records based on BP control status (within target range or elevated) or used color coding for easier interpretation [13,14,29,42,50,52,54]. A quarter of the computerized CDSSs displayed averages of the readings (5/21, 24%) [13,32,39,42,54] and recommendations for medication changes (5/21, 24%) [26,31,33,39,42] (Table S2 in Multimedia Appendix 3).

Of the 12 noncomputerized CDSSs, 3 (25%) triaged data based on predefined criteria or color-coded charts [41,43,53]. Half (6/12, 50%) allowed health care professionals to view the HBP records on a dashboard [28,30,40,41,44,53]. Only one-third (4/12, 33%) proactively recommended medication adjustments [23,36,44,48] (Table S3 in Multimedia Appendix 3).

### Outcomes

#### Adjustments in Antihypertensive Medications

Of the 18 studies that reported adjustments in antihypertensive medications, 16 (89%) reported adjustments made by physicians, with substantial changes in the intervention group observed in 90% (9/10) of the computerized CDSS studies [26,32-35,39,46,47,50] (Table S4 in Multimedia Appendix 3) and 89% (7/8) of the noncomputerized CDSS studies [28,30,36,38,41,43,44] (Table S5 in Multimedia Appendix 3).

#### Improvement in BP Control

Most of the studies (25/29, 86%) [23,25,28,29,31,33-39,41-43,45-53] showed improved BP control. Slightly more than half of these studies (15/29, 52%) were randomized controlled trials [23,28,32-35,38,39,41-43,45-48].

Nearly all computerized CDSSs that measured changes in BP (17/18, 94%) showed improvement [13,29,31-35,37,39, 42,45-47,49-52] (Table S4 in Multimedia Appendix 3). Of the noncomputerized studies that measured changes in BP control, most (8/11, 72%) reported an improvement [25,28,36,41,43,48,53], while the rest (3/11, 27%) found no improvement with the use of a CDSS [23,27,44] (Table S5 in Multimedia Appendix 3).

#### User Experiences

Of the 33 studies, 12 (36%) reported patient and clinician experiences [14,29,30,32,33,35,37,45,50,52-54].

Patients’ positive experiences with the remote monitoring models due to convenience and motivation led to improved self-management and a sense of security [14,29,30,53]. Patients appreciated the ease of use and clear data presentation of digital platforms, which also fostered better communication with health care providers [14,29,30,53,54]. However, some patients experienced challenges with data entry and were concerned about data accuracy with computerized CDSSs [53]. Technical issues, usability challenges, technology anxiety, and limited data access were also reported with computerized CDSSs [14].

Clinicians valued the digital platforms’ clear data visualization and time-saving aspects, which allowed them to manage their workload by freeing up time to manage urgent matters and improve interactions with patients [14,53]. They also reported positive experiences with workflow integration [29]. Clinicians faced usability challenges and data access limitations with certain CDSSs [14].

### CDSS Implementation

#### Patient Education

Patient education was provided in all studies that used noncomputerized CDSSs (12/12, 100%) [23,25,27,28,30,36,38,41,43,44,48,53] and in more than half of the studies that used computerized CDSSs (12/21, 57%) [13,26,29,32-35,39,42,47,49,50]. The education focused on ensuring that patients could accurately measure and record their BP, with emphasis on the use of digital tools and telemonitoring systems. Some of the studies included both spoken and written instructions on BP self-measurement and device use (3/33, 9%) [13,39,49], while others provided educational booklets (1/33, 3%) [47] or online resources such as demonstration videos (1/33, 3%) [42]. In 4 (33%) of the 12 computerized CDSS studies that provided patient education, patients were assisted in setting up accounts and shown how to upload BP data [13,32,42,49]. Of the 24 studies that provided patient education, 3 (13%) stated that instructions on HBP monitoring were provided without detailing specifics [26,27,36]. Some of the studies (6/24, 25%) included practical training sessions to ensure that patients could use the devices correctly [29,33-35,49,50]. Training was provided by health care professionals, including pharmacists (3/24, 13%), nurses (2/24, 8%), laboratory personnel (1/24, 4%), or study coordinators (2/24, 8%) [23,28,29,33-35,38,43,48,53].

#### Clinician Training

Clinician training was provided in 1 (5%) of the 21 computerized CDSS studies and in 3 (25%) of the 12 noncomputerized CDSS studies [28,44,53].

## Discussion

### Principal Findings

This review highlighted a significant gap in the use of theoretical frameworks in CDSS development. Many CDSSs relied primarily on expert consensus rather than being grounded in evidence-based hypertension guidelines. In addition, the integration of CDSSs with manual BP recording and uploading was limited. There was also a disparity in the reported outcomes in the included studies.

### Comparisons With Prior Work

Similar to our study, a systematic review by Thompson et al [55] on the effect of CDSSs on nurses and allied health professionals highlighted the absence of theoretical frameworks in CDSS development and implementation; none of the studies explicitly described a theory for developing CDSSs. This lack of transparency is critical because it impacts the system’s effectiveness and successful implementation. Khong et al [56] highlighted that the lack of theoretical frameworks in CDSS development can result in poor usability and limited consideration of context, leading to poor adoption rates among health care providers. Implementation barriers are also more likely to arise, as theoretical frameworks often identify and mitigate potential challenges [57]. A systematic approach is essential to ensure utility and usability; integrating decision science and software engineering frameworks can provide a robust foundation, ensuring that the CDSS accurately supports user decisions and offers a user-friendly interface [58].

Our review identified a single theoretical framework—a person-based approach—that attempts to gain a deep understanding of users’ psychosocial context and their views on the behavior change promoted by the intervention [42]. This approach, described by Yardley et al [59], guides intervention development using principles rooted in self-determination theory, which posits that intrinsic motivation is strengthened by supporting autonomy, competence, and perceived relatedness. While the person-based approach emphasizes user experience, it may lack the methodological rigor of decision science and software engineering frameworks. It may also overlook critical aspects such as cognitive load and algorithmic transparency, which are essential for ensuring utility and trust. To develop CDSSs that are both user centered and technically robust, future work should integrate frameworks such as software development life cycle and implementation frameworks. However, given the complexity of clinical settings and the evolving use of intelligent systems, relying on a single framework may prove insufficiently inclusive [60]. These frameworks and models should be adapted and tailored to specific contexts to ensure that they address the unique needs and challenges of each clinical setting [61].

As identified by our study, the rule-based method is widely used in CDSSs to guide clinical decision-making [62]. A systematic review of the effects of computerized CDSSs on nursing and allied health professional performance and patient outcomes highlighted the increased use of if-then rules in the 35 CDSSs examined [55]. Knowledge-based CDSSs offer transparency and consistency through predefined rules, but they require regular updates and may lack flexibility. By contrast, nonknowledge-based CDSSs, which use machine learning, adapt to evolving medical knowledge and handle large datasets but suffer from interpretability issues and computational complexity (black boxes) [15].

A hybrid approach combining knowledge-based and nonknowledge-based systems may be more suitable for hypertension management. This hybrid model can leverage the interpretability of knowledge-based systems and the adaptability of nonknowledge-based systems, potentially enhanced by explainable artificial intelligence and continuous learning mechanisms. Such a hybrid approach can provide transparency and improve trust and adoption among clinicians [15]. Sim et al [63] proposed using an “evidence-adaptive CDSS,” a subclass of CDSSs that continuously updates its clinical knowledge base to reflect the latest evidence from the literature and practice-based sources; for example, a CDSS for hypertension treatment is considered evidence adaptive if its knowledge base is grounded in current evidence and its recommendations are regularly updated to incorporate new research findings. This flexibility allows the CDSS to readily include updates and revisions to guidelines, ensuring that recommendations remain current and aligned with the latest evidence.

Many of the CDSSs (20/33, 61%) in the included studies relied on expert consensus rather than established hypertension guidelines to develop rules and algorithms. Thompson et al also highlighted the unclear evidential basis for the rules and protocols used in the CDSSs [55]. This trend could lead to variations in clinical practice and potentially undermine the standardization of hypertension management. Ensuring that CDSSs are aligned with approved robust evidence and clinically validated knowledge is crucial for maintaining the quality and consistency of care as well as enhanced outcomes [64,65]. Lobach et al [66] and Arditi et al [67] reported improved outcomes with evidence-based decision support. Given the dynamically changing evidence base, CDSSs should be designed for adaptability and agility. When evidence is lacking or inconclusive, such as with HBP targets for older adults or unique patient characteristics not fully covered in guidelines, expert opinion may be necessary to guide clinical decisions. However, when expert opinion is used, it is essential to highlight this within the CDSS to promote transparency and shared decision-making. Clinicians should be aware of the basis for the recommendations, enabling them to engage in informed discussions with patients and tailor treatment plans to individual needs and preferences [68]. This approach ensures that patients are actively involved in their care, fostering shared decision-making.

In addition, it was found that the integration of CDSSs with manual BP recording and uploading was limited. Ideally, to improve the accuracy and validity of the captured HBP records, automatic recording and uploading of the HBP readings from the BP monitoring device is the preferred method. However, patients often report technology anxiety and face difficulties with data entry [14,52]. Due to these limitations, a significant portion of patients, especially older adults with a higher prevalence of hypertension, still largely depend on manual BP recordings. Nevertheless, without proper CDSSs, manually recorded BP readings may not be accurately integrated into patient records, leading to incomplete or incorrect data being used for clinical decisions [8]. In addition, manual data entry is prone to human error, and without robust CDSSs, these errors can go unnoticed, affecting the quality of patient care. Finally, without comprehensive CDSSs, the analysis of BP data may be inconsistent, making it challenging to identify trends or patterns that could inform better treatment strategies [8,9]. These challenges underscore the need for a more seamless integration of digital tools with traditional methods to ensure the accuracy and usability of the collected data [69]. Thus, future studies should evaluate CDSSs that can effectively integrate and analyze manually and automatically recorded HBP readings, ensuring safe, accurate, and patient-centered care.

### Limitation of Evidence

The included studies exhibited significant heterogeneity, particularly in CDSS design, which either targeted clinicians exclusively, or incorporated patient interfaces for clinician interaction. The use of CDSSs also varied considerably across the studies, including differences in the targeted clinicians (nurses, pharmacists, and physicians), output types (ranging from simple alerts to specific medication recommendations), and action taken (eg, antihypertensive titration and patient counseling). Furthermore, the reported outcomes were diverse, encompassing BP improvement, antihypertensive titration, health care use, and user experiences. The 15 randomized controlled trials that reported outcomes on BP improvement and antihypertensive titrations also demonstrated substantial heterogeneity across patient populations, health care professionals, CDSS types, concurrent interventions, and reported outcomes, which precluded a detailed quantitative synthesis of effect sizes. Furthermore, these outcomes were influenced by a combination of interventions rather than the CDSS alone. Consequently, outcomes were summarized as positive or negative trends instead of precise effect magnitudes, and these results should be interpreted with caution.

### Future Directions

Future studies require a thorough process evaluation of CDSSs for decision-making, particularly in medication adjustment. This will include scrutinizing the CDSS’s data input as well as the specific patient information used and its presentation to clinicians, followed by analyzing clinician interactions, including the time spent using the system, their perceptions of its usability and usefulness, and the frequency of recommendation overrides. The decision-making process must be examined, focusing on how CDSSs influence clinicians’ choices, the factors determining recommendation adherence, and the impact on workflow. Measurable indicators such as CDSS use metrics, changes in prescribing patterns, and adherence to clinical guidelines provide quantitative data. At the same time, qualitative methods such as interviews and observations offer insights into clinician and patient experiences. Medication adjustments, timeliness, and consistency with best practices could be tracked alongside patient outcomes such as BP changes and medication adherence. When patient interaction is incorporated, its impact on their understanding and adherence can be evaluated. By capturing data on CDSS input, clinician interaction, decision-making processes, medication adjustments, and patient outcomes through both quantitative and qualitative methods, researchers could gain a comprehensive understanding of how CDSSs influence medication adjustment. This approach goes beyond simple outcome measures and helps to identify areas for improvement and refine CDSS effectiveness.

### Limitations of the Scoping Review

Meta-analyses were not conducted because of the significant heterogeneity in CDSS types and reported outcomes. Given that most of the studies were randomized controlled trials, a future systematic review, potentially including a meta-analysis, may be warranted to evaluate outcomes associated with specific CDSS types. The proposed CDSS-hypertension checklist may help standardize reporting in future CDSS studies among patients with hypertension, thereby facilitating meta-analyses.

### Conclusions

Overall, while the use of a CDSS has the potential to enhance hypertension management, our review identified gaps in CDSS development and implementation, including the absence of theoretical frameworks guiding the development process and minimal adherence to established guidelines in handling HBP data. Moreover, the integration of CDSSs with manual BP recording and data uploading was limited. These findings underscore the need for more rigorous and theory-informed development of CDSS tools, alongside greater standardization in data processing and integration workflows, to support hypertension management.

## Appendix

Multimedia Appendix 1 Database search strings used for PubMed, Embase and Scopus.

Multimedia Appendix 2 Clinical Decision Support System – Hypertension (CDSS-HTN) checklist.

Multimedia Appendix 3 Detailed characteristics, data processing sources, clinical decision support system features and outcomes of the studies included in the scoping review.

Multimedia Appendix 4 PRISMA-ScR checklist.

## Abbreviations

BP: blood pressure
CDSS: clinical decision support system
HBP: home blood pressure
MeSH: Medical Subject Headings
PRISMA-ScR: Preferred Reporting Items for Systematic Reviews and Meta-Analyses extension for Scoping Reviews

## Data Availability

The data analyzed in the study are available in the main manuscript and Multimedia Appendices 2 and 3. Additional data associated with the study will be available from the corresponding author on reasonable request.
