# Supplementary material for: Clinical Decision Support Systems Using Home Blood Pressure Readings to Manage Patients With Hypertension: Scoping Review
Source: J Med Internet Res. 2025 Oct 3;27:e75551. doi: 10.2196/75551 (PMC12534771; doi:10.2196/75551)
Supplement: Multimedia Appendix 1 [file jmir_v27i1e75551_app1.docx]

**PubMed**

("Expert system"[All Fields] OR "Diagnostic decision support"[All Fields] OR "Rules engine"[All Fields] OR "Inference engine"[All Fields] OR "Information systems"[All Fields] OR "point of care system"[All Fields] OR "reminder system"[All Fields] OR "reminder system*"[All Fields] OR ("reminder system"[All Fields] AND "comput*"[All Fields]) OR (("reminder systems"[MeSH Terms] OR ("reminder"[All Fields] AND "systems"[All Fields]) OR "reminder systems"[All Fields] OR ("reminder"[All Fields] AND "System"[All Fields]) OR "reminder system"[All Fields]) AND "comput*"[All Fields]) OR "alert system"[All Fields] OR "Dashboard"[All Fields] OR "Data visualisation"[All Fields] OR "Data reports"[All Fields] OR "Personal Health Record"[All Fields] OR "Medical Order Entry Systems"[All Fields] OR "Decision support"[All Fields] OR "Decision Support Techniques"[All Fields] OR "decision making computer assisted"[All Fields] OR "decision support systems clinical"[All Fields] OR "Electronic Medical Record"[All Fields] OR "Electronic Health Record"[All Fields] OR "Tool"[All Fields] OR "Aid"[All Fields] OR "comput* decision aid"[All Fields] OR "comput* decision making"[All Fields] OR "clinical decision support aid*"[All Fields] OR "System"[All Fields] OR "Technique"[All Fields] OR "Guide"[All Fields] OR "Care pathways"[All Fields] OR (("decision support systems, clinical"[MeSH Terms] OR ("decision"[All Fields] AND "support"[All Fields] AND "systems"[All Fields] AND "clinical"[All Fields]) OR "clinical decision support systems"[All Fields] OR ("clinical"[All Fields] AND "decision"[All Fields] AND "support"[All Fields]) OR "clinical decision support"[All Fields]) AND "guideline*"[All Fields]) OR "CDSS"[All Fields] OR "CCDSS"[All Fields] OR "CCDS"[All Fields] OR "Clinical decision support system"[All Fields] OR "clinical decision support system*"[All Fields] OR "comput* assisted diagnosis"[All Fields] OR "diagnosis computer assisted"[All Fields] OR ("computer assisted"[All Fields] AND ("drug therapy"[MeSH Subheading] OR ("drug"[All Fields] AND "therapy"[All Fields]) OR "drug therapy"[All Fields] OR "drug therapy"[MeSH Terms])) OR ("computer assisted"[All Fields] AND ("diagnosable"[All Fields] OR "diagnosi"[All Fields] OR "diagnosis"[MeSH Terms] OR "diagnosis"[All Fields] OR "diagnose"[All Fields] OR "diagnosed"[All Fields] OR "diagnoses"[All Fields] OR "diagnosing"[All Fields] OR "diagnosis"[MeSH Subheading])) OR (("computability"[All Fields] OR "computable"[All Fields] OR "computating"[All Fields] OR "computation"[All Fields] OR "computational"[All Fields] OR "computations"[All Fields] OR "compute"[All Fields] OR "computed"[All Fields] OR "computer s"[All Fields] OR "computers"[MeSH Terms] OR "computers"[All Fields] OR "computer"[All Fields] OR "computes"[All Fields] OR "computing"[All Fields] OR "computional"[All Fields]) AND ("diagnosable"[All Fields] OR "diagnosi"[All Fields] OR "diagnosis"[MeSH Terms] OR "diagnosis"[All Fields] OR "diagnose"[All Fields] OR "diagnosed"[All Fields] OR "diagnoses"[All Fields] OR "diagnosing"[All Fields] OR "diagnosis"[MeSH Subheading])) OR ("computer assisted"[All Fields] AND ("decision"[All Fields] OR "decision s"[All Fields] OR "decisions"[All Fields] OR "decisive"[All Fields] OR "decisively"[All Fields])) OR (("computability"[All Fields] OR "computable"[All Fields] OR "computating"[All Fields] OR "computation"[All Fields] OR "computational"[All Fields] OR "computations"[All Fields] OR "compute"[All Fields] OR "computed"[All Fields] OR "computer s"[All Fields] OR "computers"[MeSH Terms] OR "computers"[All Fields] OR "computer"[All Fields] OR "computes"[All Fields] OR "computing"[All Fields] OR "computional"[All Fields]) AND ("drug therapy"[MeSH Subheading] OR ("drug"[All Fields] AND "therapy"[All Fields]) OR "drug therapy"[All Fields] OR "drug therapy"[MeSH Terms])) OR (("computability"[All Fields] OR "computable"[All Fields] OR "computating"[All Fields] OR "computation"[All Fields] OR "computational"[All Fields] OR "computations"[All Fields] OR "compute"[All Fields] OR "computed"[All Fields] OR "computer s"[All Fields] OR "computers"[MeSH Terms] OR "computers"[All Fields] OR "computer"[All Fields] OR "computes"[All Fields] OR "computing"[All Fields] OR "computional"[All Fields]) AND ("assistances"[All Fields] OR "assistant s"[All Fields] OR "assistants"[All Fields] OR "assisted"[All Fields] OR "assisting"[All Fields] OR "assistive"[All Fields] OR "dental assistants"[MeSH Terms] OR ("dental"[All Fields] AND "assistants"[All Fields]) OR "dental assistants"[All Fields] OR "assistant"[All Fields] OR "helping behavior"[MeSH Terms] OR ("helping"[All Fields] AND "behavior"[All Fields]) OR "helping behavior"[All Fields] OR "assist"[All Fields] OR "assistance"[All Fields] OR "assists"[All Fields]) AND "therap*"[All Fields]) OR "therapy computer assisted"[All Fields] OR (("computability"[All Fields] OR "computable"[All Fields] OR "computating"[All Fields] OR "computation"[All Fields] OR "computational"[All Fields] OR "computations"[All Fields] OR "compute"[All Fields] OR "computed"[All Fields] OR "computer s"[All Fields] OR "computers"[MeSH Terms] OR "computers"[All Fields] OR "computer"[All Fields] OR "computes"[All Fields] OR "computing"[All Fields] OR "computional"[All Fields]) AND ("therapeutics"[MeSH Terms] OR "therapeutics"[All Fields] OR "therapies"[All Fields] OR "therapy"[MeSH Subheading] OR "therapy"[All Fields] OR "therapy s"[All Fields] OR "therapys"[All Fields])) OR (("computability"[All Fields] OR "computable"[All Fields] OR "computating"[All Fields] OR "computation"[All Fields] OR "computational"[All Fields] OR "computations"[All Fields] OR "compute"[All Fields] OR "computed"[All Fields] OR "computer s"[All Fields] OR "computers"[MeSH Terms] OR "computers"[All Fields] OR "computer"[All Fields] OR "computes"[All Fields] OR "computing"[All Fields] OR "computional"[All Fields]) AND ("decision"[All Fields] OR "decision s"[All Fields] OR "decisions"[All Fields] OR "decisive"[All Fields] OR "decisively"[All Fields])) OR ("comput*"[All Fields] AND (("decision"[All Fields] OR "decision s"[All Fields] OR "decisions"[All Fields] OR "decisive"[All Fields] OR "decisively"[All Fields]) AND "support*"[All Fields])) OR "Computerised"[All Fields] OR "Computerized"[All Fields] OR "computer assisted"[All Fields] OR "Computer-based"[All Fields] OR "Computer-aided"[All Fields] OR "Online"[All Fields] OR "Web-based"[All Fields] OR "Digital"[All Fields] OR "Electronic"[All Fields] OR "electronic*"[All Fields] OR "Email based"[All Fields])

AND

("Self blood pressure measurement"[All Fields] OR (("ego"[MeSH Terms] OR "ego"[All Fields] OR "self"[All Fields]) AND ("blood pressure determination"[MeSH Terms] OR ("blood"[All Fields] AND "pressure"[All Fields] AND "determination"[All Fields]) OR "blood pressure determination"[All Fields])) OR "Self blood pressure monitoring"[All Fields] OR "Home blood pressure measurement"[All Fields] OR "Home blood pressure monitoring"[All Fields] OR "Blood pressure telemonitoring"[All Fields] OR (("smartphone"[MeSH Terms] OR "smartphone"[All Fields] OR "smartphones"[All Fields] OR "smartphone s"[All Fields]) AND ("monitor"[All Fields] OR "monitor s"[All Fields] OR "monitorable"[All Fields] OR "monitored"[All Fields] OR "monitoring"[All Fields] OR "monitoring s"[All Fields] OR "monitorings"[All Fields] OR "monitorization"[All Fields] OR "monitorize"[All Fields] OR "monitorized"[All Fields] OR "monitors"[All Fields]) AND ("blood pressure"[MeSH Terms] OR ("blood"[All Fields] AND "pressure"[All Fields]) OR "blood pressure"[All Fields] OR "blood pressure determination"[MeSH Terms] OR ("blood"[All Fields] AND "pressure"[All Fields] AND "determination"[All Fields]) OR "blood pressure determination"[All Fields] OR "arterial pressure"[MeSH Terms] OR ("arterial"[All Fields] AND "pressure"[All Fields]) OR "arterial pressure"[All Fields])) OR (("remote"[All Fields] OR "remotely"[All Fields] OR "remoteness"[All Fields] OR "remotes"[All Fields]) AND ("monitor"[All Fields] OR "monitor s"[All Fields] OR "monitorable"[All Fields] OR "monitored"[All Fields] OR "monitoring"[All Fields] OR "monitoring s"[All Fields] OR "monitorings"[All Fields] OR "monitorization"[All Fields] OR "monitorize"[All Fields] OR "monitorized"[All Fields] OR "monitors"[All Fields]) AND ("blood pressure"[MeSH Terms] OR ("blood"[All Fields] AND "pressure"[All Fields]) OR "blood pressure"[All Fields] OR "blood pressure determination"[MeSH Terms] OR ("blood"[All Fields] AND "pressure"[All Fields] AND "determination"[All Fields]) OR "blood pressure determination"[All Fields] OR "arterial pressure"[MeSH Terms] OR ("arterial"[All Fields] AND "pressure"[All Fields]) OR "arterial pressure"[All Fields])) OR (("wearable electronic devices"[MeSH Terms] OR ("wearable"[All Fields] AND "Electronic"[All Fields] AND "devices"[All Fields]) OR "wearable electronic devices"[All Fields] OR ("wearable"[All Fields] AND "technology"[All Fields]) OR "wearable technology"[All Fields]) AND ("blood press monit"[Journal] OR ("blood"[All Fields] AND "pressure"[All Fields] AND "monitoring"[All Fields]) OR "blood pressure monitoring"[All Fields])) OR (("biometric"[All Fields] OR "biometrical"[All Fields] OR "biometrically"[All Fields] OR "biometry"[MeSH Terms] OR "biometry"[All Fields] OR "biometrics"[All Fields]) AND ("blood press monit"[Journal] OR ("blood"[All Fields] AND "pressure"[All Fields] AND "monitoring"[All Fields]) OR "blood pressure monitoring"[All Fields])))

**Embase** (restricted source to Embase)

('expert system' OR 'diagnostic decision support' OR 'rules engine' OR 'inference engine' OR 'information systems' OR 'point of care system' OR 'reminder system' OR 'reminder system*' OR (reminder AND system AND comput*) OR 'alert system' OR 'dashboard' OR 'data visualisation' OR 'data reports' OR 'personal health record' OR 'medical order entry systems' OR 'decision support' OR 'decision support techniques' OR 'decision making, computer-assisted' OR 'decision support systems, clinical' OR 'electronic medical record' OR 'electronic health record' OR 'tool' OR 'aid' OR 'comput* decision aid' OR 'comput* decision making' OR 'clinical decision support aid*' OR 'system' OR 'technique' OR 'guide' OR 'care pathways' OR (clinical AND decision AND support AND guideline*) OR 'cdss' OR 'ccdss' OR 'ccds' OR 'clinical decision support system' OR 'clinical decision support system*' OR 'comput* assisted diagnosis' OR 'diagnosis, computer-assisted' OR ('computer assisted' AND drug AND therapy) OR ('computer assisted' AND diagnosis) OR (computer AND diagnosis) OR ('computer assisted' AND decision) OR (computer AND 'drug therapy') OR (computer AND assisted AND therap*) OR 'therapy, computer-assisted' OR (computer AND therapy) OR (computer AND decision) OR (comput* AND decision AND support*) OR 'computerised' OR 'computer-assisted' OR 'computer-based' OR 'computer-aided' OR 'online' OR 'web-based' OR 'digital' OR 'electronic' OR 'electronic*' OR 'email based')

AND

('self blood pressure measurement' OR (self AND blood AND pressure AND determination) OR 'self blood pressure monitoring' OR 'home blood pressure measurement' OR 'home blood pressure monitoring' OR 'blood pressure telemonitoring' OR (smartphone AND monitored AND blood AND pressure) OR (remote AND monitored AND blood AND pressure) OR (wearable AND technology AND blood AND pressure AND monitoring) OR (biometric AND blood AND pressure AND monitoring))

**SCOPUS**

( TITLE-ABS-KEY ( "expert system" OR "diagnostic decision support" OR "rules engine" OR "inference engine" OR "information system*" OR "point of care system" OR "reminder system*" OR "reminder system W/3 comput*" OR "alert system" OR "dashboard" OR "data visualization" OR "data report*" OR "personal health record" OR "medical order entry system*" OR "decision support" OR "decision support technique*" OR "decision making W/3 computer-assisted" OR "decision support system* W/3 clinical" OR "electronic medical record" OR "electronic health record" OR "tool" OR "aid" OR "comput* decision aid" OR "comput* decision making" OR "clinical decision support aid*" OR "system" OR "technique" OR "guide" OR "care pathway*" OR "clinical decision support guideline*" OR "CDSS" OR "CCDSS" OR "CCDS" OR "clinical decision support system*" OR "comput* assisted diagnosis" OR "diagnosis W/3 computer-assisted" OR "computer-assisted W/3 drug therapy" OR "computer-assisted W/3 diagnosis" OR "computer W/3 diagnosis" OR "computer-assisted W/3 decision" OR "computer W/3 drug-therapy" OR "computer assisted therap*" OR "therapy W/3 computer" OR "computer W/3 decision" OR "comput* W/3 decision support*" OR "computerised" OR "computerized" OR "computer-assisted" OR "computer-based" OR "computer-aided" OR "online" OR "web-based" OR "digital" OR "electronic*" OR "email based" ) )

AND

( TITLE-ABS-KEY ( "self blood pressure measurement" OR "self blood pressure determination" OR "self blood pressure monitoring" OR "home blood pressure measurement" OR "home blood pressure monitoring" OR "blood pressure telemonitoring" OR "smartphone monitored blood pressure" OR "remote monitored blood pressure" OR "wearable technology blood pressure monitoring" OR "biometric blood pressure monitoring" ) )
