# Supplementary material for: Clinical Decision Support Systems Using Home Blood Pressure Readings to Manage Patients With Hypertension: Scoping Review
Source: J Med Internet Res. 2025 Oct 3;27:e75551. doi: 10.2196/75551 (PMC12534771; doi:10.2196/75551)
Supplement: Multimedia Appendix 2 [file jmir_v27i1e75551_app2.docx]

Supplementary file 1. Clinical Decision Support System – Hypertension (CDSS-HTN) checklist

| **Item number** | **Checklist item** | **Page number where section is reported** |
| --- | --- | --- |
| **TITLE** | | |
| 1 | Identify manuscript as a study evaluating clinical decision support system among patients with hypertension |  |
| **ABSTRACT** | | |
| 2 | Summarise the background, methodology, key research findings and conclusion of study |  |
| **INTRODUCTION** | | |
| 3 | Describe the reasons for conducting the study and contextualise it in relation to current literature |  |
| 4 | Describe the objective or research question for the study |  |
| **METHODOLOGY** | | |
| 5 | Study design  - Describe the study methodology |  |
| 6 | Setting  – Describe the study setting, start and end date of study, follow-up duration |  |
| 7 | Participants  - Describe the inclusion and exclusion criteria for study participants and methodology of selecting participants  - Describe the sample size |  |
| 8 | Design and function of clinical decision support system   - Describe the type of clinical decision support system - Describe the theoretical framework used for design of the clinical decision support system - Describe the data capture of the clinical decision support system with regards to internal and external data sources, and data collection methods - Describe the data processing layer of the clinical decision support system in relation to the knowledge or non-knowledge base - Describe the data output layer of the clinical decision support system in relation to presentation of results, recommendations, and clinical decision |  |
| 9 | Outcomes   - Describe all clinical, patient and healthcare related outcomes collected - Describe feedback from patients and doctors related to use of clinical decision support system - Describe how outcomes were assessed and provide details pertaining to instruments used (if relevant) for assessment of outcomes |  |
| 10 | Statistics   - Describe all statistical methods used in the study - Describe the handling of missing data |  |
| **RESULTS** | | |
| 11 | Participants   - Provide the number of participants recruited and reasons for drop-out - Describe the characteristics of study participants |  |
| 12 | Outcomes   - Summarise and report outcomes collected in study |  |
| **DISCUSSION** | | |
| 13 | Key results  Provide a summary of study results in relation to the study objective |  |
| 14 | Interpretation   - Provide the interpretation of study results and discuss it in relation to other studies and evidence available in the literature |  |
| 15 | Limitations   - Describe the limitations of the study |  |
| **FUNDING** | | |
| 16 | Funding  Provide the funding source of the study |  |
