# Supplementary material for: Clinical Decision Support Systems Using Home Blood Pressure Readings to Manage Patients With Hypertension: Scoping Review
Source: J Med Internet Res. 2025 Oct 3;27:e75551. doi: 10.2196/75551 (PMC12534771; doi:10.2196/75551)
Supplement: Multimedia Appendix 3 [file jmir_v27i1e75551_app3.docx]

**Table S1: Characteristics of the studies included**

| **S#** | **1^st^ author and study year** | **Title** | **Country** | **Study design** | **Study setting** | **Total number of participants** | **Type of CDSS** |
| --- | --- | --- | --- | --- | --- | --- | --- |
|  | Tobe 2008 [48] | IMPPACT: Investigation of medical professionals and patients achieving control together | Canada | Randomised controlled trial | Primary Care/ GP; Cardiology outpatients | 323 (53 physicians, 270 patients) | Non-computerised |
|  | Parati 2009 [46] | Home blood pressure telemonitoring improves hypertension control in general practice. the TeleBPCare study | Italy | Randomised controlled trial | Primary Care/ GP | 329 | Computerised |
|  | Crowley 2011 [26] | Treatment intensification in a hypertension telemanagement trial: Clinical inertia or good clinical judgment? | United States | Randomised controlled trial | Primary Care/ GP | 296 | Computerised |
|  | Margolis 2012 [33] | Effect of Home Blood Pressure Telemonitoring and Pharmacist Management On Blood Pressure Control: The HyperLink Cluster Randomized Trial | United States | Randomised controlled trial | Primary Care/ GP; multispecialty practice | 450 | Computerised |
|  | Xiao 2019 [53] | Home blood pressure monitoring by a mobile-based model in chongqing, China: A feasibility study | China | Qualitative research | Primary Care/ GP | 22 (20 patients and 2 providers) | Non-computerised |
|  | Teo 2021 [14] | Effects of technology-enabled blood pressure monitoring in primary care: A quasi-experimental trial | Singapore | Non-randomised experimental study | Primary Care/ GP | 242 | Computerised |
|  | Mehos 2000 [36] | Effect of pharmacist intervention and initiation of home blood pressure monitoring in patients with uncontrolled hypertension | United States | Randomised controlled trial | Primary Care/ GP | 36 | Non-computerised |
|  | Neumann 2011 [45] | Blood pressure telemonitoring is useful to achieve blood pressure control in inadequately treated patients with arterial hypertension | Germany | Randomised controlled trial | Not specified. | 60 | Computerised |
|  | Margolis 2018 [35] | Long-term Outcomes of the Effects of Home Blood Pressure Telemonitoring and Pharmacist Management on Blood Pressure Among Adults With Uncontrolled Hypertension: Follow-up of a Cluster Randomized Clinical Trial. | United States | Randomised controlled trial | Primary Care/ GP | 450 | Computerised |
|  | Moreira 2023 [44] | Optimizing Hypertension Treatment in Older Patients Through Home Blood Pressure Monitoring by Pharmacists in Primary Care: The MINOR Clinical Trial. | Brazil | Randomised controlled trial | Primary Care/ GP | 322 | Non-computerised |
|  | Margolis 2015 [34] | A Successful Multifaceted Trial to Improve Hypertension Control in Primary Care: Why Did it Work? | United States | Randomised controlled trial | Primary Care/ GP | 403 | Computerised |
|  | Egede 2021 [27] | Randomized controlled trial of technology-assisted case management in low-income adults with type 2 diabetes: Effect on quality of life and blood pressure. | United States | Randomised controlled trial | Primary Care/ GP | 113 | Non-computerised |
|  | Rinfret 2009 [47] | The impact of a multidisciplinary information technology-supported program on blood pressure control in primary care. | Canada | Randomised controlled trial | Primary Care/ GP | 223 | Computerised |
|  | Peters 2017 [37] | Assessing the Utility of a Novel SMS- and Phone-Based System for Blood Pressure Control in Hypertensive Patients: Feasibility Study. | United States | Quality improvement | Primary Care/ GP | 174 | Computerised |
|  | Asayama 2012 [39] | Cardiovascular outcomes in the first trial of antihypertensive therapy guided by self-measured home blood pressure. | Japan | Randomised controlled trial | Primary Care/ GP | 3518 | Computerised |
|  | Magid 2013 [32] | A pharmacist-led, American Heart Association Heart360 Web-enabled home blood pressure monitoring program. | United States | Randomised controlled trial | Primary Care/ GP | 348 | Computerised |
|  | Willis 2023 [51] | Real-world long-term effects on blood pressure and other cardiovascular risk factors for patients in Computerised therapeutics. | Sweden | Cohort study | Primary Care/ GP | 7752 | Computerised |
|  | McManus 2021 [42] | Home and Online Management and Evaluation of Blood Pressure (HOME BP) using a Computerised intervention in poorly controlled hypertension: randomised controlled trial. | United Kingdom | Randomised controlled trial | Primary Care/ GP | 698 (76 GPs, 622 patients) | Computerised |
|  | Hoppe 2019 [30] | Telehealth with remote blood pressure monitoring for postpartum hypertension: A prospective single-cohort feasibility study. | United States | Cohort study | Hospital | 55 | Non-computerised |
|  | McKinstry 2013 [41] | Telemonitoring based service redesign for the management of uncontrolled hypertension: multicentre randomised controlled trial. | Scotland | Randomised controlled trial | Primary Care/ GP | 401 | Non-computerised |
|  | Teo 2023 [52] | Implementation and use of technology-enabled blood pressure monitoring and teleconsultation in Singapore's primary care: a qualitative evaluation using the socio-technical systems approach. | Singapore | Qualitative research | Primary Care/ GP | 21 (13 patients and 8 Clinician) | Computerised |
|  | McManus 2018 [43] | Efficacy of self-monitored blood pressure, with or without telemonitoring, for titration of antihypertensive medication (TASMINH4): an unmasked randomised controlled trial. | United Kingdom | Randomised controlled trial | Primary Care/ GP | 1182 (142 practices) | Non-computerised |
|  | Gupta 2023 [29] | Implementing a home-based virtual hypertension programme-a pilot feasibility study. | United States | Mixed method: RCT + qualitative | Primary Care/ GP | 42 (Patients: 31 Pharmacists: 5 Physicians: 6) | Computerised |
|  | Carrera 2016 [54] | BPcontrol. A Mobile App to Monitor Hypertensive Patients. | Not reported | Cross sectional study | Community | Pilot study: 33 Usability: 20 | Computerised |
|  | Green 2008 [28] | Effectiveness of home blood pressure monitoring, Web communication, and pharmacist care on hypertension control: a randomized controlled trial. | United States | Randomised controlled trial | Primary Care/ GP; Hospital | 778 | Non-computerised |
|  | Blood 2023 [24] | Results of a Remotely Delivered Hypertension and Lipid Program in More Than 10â€¯000 Patients Across a Diverse Health Care Network. | United States | Non-randomised experimental study | Primary Care/ GP | 10,803 | Non-computerised |
|  | Broege 2001 [25] | Management of hypertension in the elderly using home blood pressures. | United States | Randomised controlled trial | Primary Care/ GP; Hypertension Center of Weill Medical College of Cornell University | 40 | Non-computerised |
|  | Jackson 2012 [31] | Racial differences in the effect of a telephone-delivered hypertension disease management program. | United States | Randomised controlled trial | Primary Care/ GP | 573 | Computerised |
|  | Kario 2021 [40] | Computerised therapeutics for essential hypertension using a smartphone application: A randomized, open-label, multicenter pilot study. | Japan | Randomised controlled trial | Primary Care/ GP | 146 | Computerised |
|  | Lv 2017 [13] | Personalized Hypertension Management Using Patient-Generated Health Data Integrated With Electronic Health Records (EMPOWER-H): Six-Month Pre-Post Study. | United States | Cohort study | Primary Care/ GP | 149 | Computerised |
|  | Ralston 2014 [38] | Home blood pressure monitoring, secure electronic messaging and medication intensification for improving hypertension control: a mediation analysis. | United States | Randomised controlled trial | Integrated health care system | 258 | Non-computerised |
|  | Wijkman 2020 [50] | A pilot study of hypertension management using a telemedicine treatment approach. | Sweden | Cohort study | Community | 172 | Computerised |
|  | Lu 2019 [49] | Effect of home telehealth care on blood pressure control: A public healthcare centre model. | Taiwan | Cohort study | Community | 432 | Computerised |
| CDSS: Clinical decision support system; GP: General Practitioner | | | | | | | |

| **Table S2: Computerised Clinical Decision Support Systems sources for data processing and their features.** | | | | | | |
| --- | --- | --- | --- | --- | --- | --- |
| **Study ID** | **Source of data processing algorithm** | **Output features of the Computerised CDSS** | | | | |
|  |  | **Alert triggered** | **Dashboard** | **Data prioritization** | **Provide average reading** | **Proactive recommendation** |
| Teo 2021, Teo 2023 [14, 52] | Expert consensus | + | + | + | - | - |
| Parati 2009 [46] | Expert consensus | + | + | - | - | - |
| Crowley 2011 [26] | Hypertension guideline based | + | - | - | - | + |
| Margolis 2012, 2015, 2018 [33-35] | Expert consensus | + | + | - | - | + |
| Neumann 2011 [45] | Expert consensus | + | + | - | - | - |
| Rinfret 2009 [47] | Expert consensus | + | - | - | - | - |
| Peters 2017 [37] | Expert consensus; Hypertension guideline based | + | - | - | - | - |
| Asayama 2012 [39] | Hypertension guideline based | + | + | - | + | + |
| Magid 2013 [32] | Expert consensus | + | + | - | + | - |
| Willis 2023 [51] | Hypertension guideline based | + | - | - | - | - |
| McManus 2021 [42] | Hypertension guideline based | + | + | + | + | + |
| Gupta 2023 [29] | Expert consensus | + | + | + | - | - |
| Carrera 2016 [26] | Expert consensus | + | - | + | + | - |
| Jackson 2012 [31] | Expert consensus | + | - | - | - | + |
| Lv 2017 [13] | Hypertension guideline based | - | + | + | + | - |
| Wijkman 2020 [50] | Expert consensus | + | - | + | - | - |
| Lu 2019 [49] | Expert consensus; Hypertension guideline based | + | - | - | - | - |
| Kario 2021 [40] | Hypertension guidelines | - | + | - | - | - |
| “+”= feature present; “-”= features not reported or absent | | | | | | |

| **Table S3: Non-computerised Clinical Decision Support Systems sources for data processing and their features.** | | | | | | |
| --- | --- | --- | --- | --- | --- | --- |
| **Study ID** | **Content (source)** | **Trigger alert** | **Dashboard** | **Data prioritization** | **Calculate average BP** | **Proactive recommendation** |
| Tobe 2008 [48] | Expert consensus | - | - | - | - | + |
| Xiao 2019 [53] | Hypertension guideline based | - | + | + | + | - |
| Mehos 2000 [36] | Expert consensus | - | - | - | + | + |
| Moreira 2023 [44] | Expert consensus | - | + | - | + | + |
| Egede 2021[27] | Expert consensus | - | + | - | - | - |
| Hoppe 2019 [30] | Expert consensus | - | + | - | - | - |
| McKinstry 2013 [41] | Hypertension guideline based | - | + | + | + | - |
| McManus 2018 [43] | Expert consensus; Hypertension guideline based | - | - | + | - | - |
| Green 2008, Ralston 2014 [28] | Expert consensus; Hypertension guideline based | - | + | - | - | - |
| Blood 2023 [24] | Expert consensus | - | - | - | + | - |
| Broege 2001 [25] | Hypertension guideline based | - | - | - | - | + |
| “+” = feature reported, “-” feature not reported | | | | | | |

| **Table S4: Outcomes reported in studies using computerised CDSS** | | | | | | | | | | |  |
| --- | --- | --- | --- | --- | --- | --- | --- | --- | --- | --- | --- |
| **Study ID** | **Improve in BP control** | **Improve HBPM** | **Improve med-cation adherence** | **Improvement in QOL** | **Adjustment in medication** | **Adverse effects** | **Reduced healthcare utilization** | **Patient experience** | **Clinician experience** | **Cost effective** |  |
| Teo 2021, Teo 2023 [14, 52] | Y | - | - | N | - | - | N | +ve/-ve | +ve/-ve | Y |  |
| Parati 2009 [46] | Y | - | Y | N | Y | - | - | - | - | N |  |
| Crowley 2011 [26] | - | - | - | - | Y | - |  |  |  |  |  |
| Margolis 2012, 2015, 2018 [33-35] | Y | Y | Y | - | Y | N | - | +ve | - | - |  |
| Neumann 2011 [45] | Y | - | - | - | N | - | - | +ve | - | - |  |
| Rinfret 2009 [47] | Y | - | Y |  | Y | N | N | - | - | - |  |
| Peters 2017 [37] | Y | - | - | - | - | - | - | +ve | - | - |  |
| Asayama 2012 [39] | Y | - | - | - | Y | - | - | - | - | - |  |
| Magid 2013 [32] | Y | Y | N | - | Y | - | N | +ve | - | - |  |
| Willis 2023 [51] | Y | - | - | - | - | - | - | - | - | - |  |
| McManus 2021 [42] | Y | - | N | N | - | N | N | - | - | Y |  |
| Gupta 2023 [29] | Y | Y | - | - | - | N | - | +ve | +ve | - |  |
| Carrera 2016 [26] | - | - | - | - | - | - | - | +ve | +ve | - |  |
| Jackson 2012 [31] | Y | - | - | - | - | - | - | - | - | - |  |
| Lv 2017 [13] | Y | - | - | N |  |  |  |  |  |  |  |
| Wijkman 2020 [50] | Y | - | - | - | Y | - | - | +ve | - | - |  |
| Lu 2019 [49] | Y | Y | - | - | - | - | - | - | - | - |  |
| Kario 2021 [40] | N | - | - | - | - | N | - | - | - | - |  |
| “Y” = Yes, “N”= No, “-“ = Not reported, “+ve” = positive experience, “-ve” = negative experience; HBPM = home BP monitoring, QOL = quality of life | | | | | | | | | | | |

| **Table S5 Outcomes reported in studies with Non-computerised CDSS** | | | | | | | | | | |  |
| --- | --- | --- | --- | --- | --- | --- | --- | --- | --- | --- | --- |
| **Study ID** | **Improve in BP control** | **Improve HBPM** | **Improve med-cation adherence** | **Improve in QOL** | **Adjustment in medication** | **Adverse effects** | **Reduced healthcare utilization** | **Patient experience** | **Clinician experience** | **Cost effective** | |
| Tobe 2008 [48] | Y | - | - | - | - | - | - | - | - | - | |
| Xiao 2019 [53] | Y | Y | - | - | - | - | - | +ve/-ve | +ve | - | |
| Mehos 2000 [36] | Y | Y | N | N | Y | - | N | - | - | - | |
| Moreira 2023 [44] | N | - | - | - | Y | - | - | - | - | - | |
| Egede 2021[27] | N | - | - | N | - | - | - | - | - | - | |
| Hoppe 2019 [30] | - | - | - | - | Y | - | - | +ve | - | - | |
| McKinstry 2013 [41] | Y | Y | N | N | Y | N | N | - | - | N | |
| McManus 2018 [43] | Y | - | N | - | Y | N | N | - | - | - | |
| Green 2008, Ralston 2014 [28] | Y | - | Y | - | Y | N | N | - | - | - | |
| Blood 2023 [24] | Y | - | - | - | - | - | - | - | - | - | |
| Broege 2001 [25] | N | - | - | - | N | - | - | - | - | - | |
| “Y”: Yes, “N”: No, “-“: not reported, +ve = positive experience, -ve; HBPM = home BP monitoring, QOL = quality of life | | | | | | | | | | | |

References:

[13] N. Lv, L. Xiao, M. L. Simmons, L. G. Rosas, A. Chan, and M. Entwistle, "Personalized Hypertension Management Using Patient-Generated Health Data Integrated With Electronic Health Records (EMPOWER-H): Six-Month Pre-Post Study," J Med Internet Res, vol. 19, no. 9, p. e311, Sep 19 2017, doi: 10.2196/jmir.7831.

[14] S. H. Teo, E. A. L. Chew, D. W. L. Ng, W. E. Tang, G. C. H. Koh, and V. H. Y. Teo, "Implementation and use of technology-enabled blood pressure monitoring and teleconsultation in Singapore's primary care: a qualitative evaluation using the socio-technical systems approach," BMC Prim Care, vol. 24, no. 1, p. 71, Mar 16 2023, doi: 10.1186/s12875-023-02014-8.

[24] A. J. Blood et al., "Results of a Remotely Delivered Hypertension and Lipid Program in More Than 10 000 Patients Across a Diverse Health Care Network," JAMA Cardiology, vol. 8, no. 1, p. 12, 2023, doi: 10.1001/jamacardio.2022.4018.

[25] P. A. Broege, G. D. James, and T. G. Pickering, "Management of hypertension in the elderly using home blood pressures," Blood Pressure Monitoring, vol. 6, no. 3, pp. 139-144, 2001. [Online]. Available: https://journals.lww.com/bpmonitoring/fulltext/2001/06000/management_of_hypertension_in_the_elderly_using.4.aspx.

[26] M. J. Crowley et al., "Treatment intensification in a hypertension telemanagement trial: clinical inertia or good clinical judgment?," Hypertension, vol. 58, no. 4, pp. 552-8, Oct 2011, doi: 10.1161/HYPERTENSIONAHA.111.174367.

[27] L. E. Egede, A. Z. Dawson, R. J. Walker, E. Garraci, and R. G. Knapp, "Randomized controlled trial of technology-assisted case management in low-income adults with type 2 diabetes: Effect on quality of life and blood pressure," J Telemed Telecare, vol. 30, no. 1, pp. 107-115, Jan 2024, doi: 10.1177/1357633X211028491.

[28] B. B. Green et al., "Effectiveness of home blood pressure monitoring, Web communication, and pharmacist care on hypertension control: a randomized controlled trial," JAMA, vol. 299, no. 24, pp. 2857-67, Jun 25 2008, doi: 10.1001/jama.299.24.2857.

[29] A. Gupta et al., "Implementing a home-based virtual hypertension programme-a pilot feasibility study," Fam Pract, vol. 40, no. 2, pp. 414-422, Mar 28 2023, doi: 10.1093/fampra/cmac084.

[30] K. K. Hoppe et al., "Telehealth with remote blood pressure monitoring for postpartum hypertension: A prospective single-cohort feasibility study," Pregnancy Hypertens, vol. 15, pp. 171-176, Jan 2019, doi: 10.1016/j.preghy.2018.12.007.

[31] G. L. Jackson et al., "Racial differences in the effect of a telephone-delivered hypertension disease management program," J Gen Intern Med, vol. 27, no. 12, pp. 1682-9, Dec 2012, doi: 10.1007/s11606-012-2138-x.

[32] D. J. Magid, K. L. Olson, S. J. Billups, N. M. Wagner, E. E. Lyons, and B. A. Kroner, "A pharmacist-led, American Heart Association Heart360 Web-enabled home blood pressure monitoring program," Circ Cardiovasc Qual Outcomes, vol. 6, no. 2, pp. 157-63, Mar 1 2013, doi: 10.1161/CIRCOUTCOMES.112.968172.

[33] K. L. Margolis et al., "Effect of home blood pressure telemonitoring and pharmacist management on blood pressure control: a cluster randomized clinical trial," JAMA, vol. 310, no. 1, pp. 46-56, Jul 3 2013, doi: 10.1001/jama.2013.6549.

[34] K. L. Margolis et al., "A Successful Multifaceted Trial to Improve Hypertension Control in Primary Care: Why Did it Work?," J Gen Intern Med, vol. 30, no. 11, pp. 1665-72, Nov 2015, doi: 10.1007/s11606-015-3355-x.

[35] K. L. Margolis et al., "Long-term Outcomes of the Effects of Home Blood Pressure Telemonitoring and Pharmacist Management on Blood Pressure Among Adults With Uncontrolled Hypertension: Follow-up of a Cluster Randomized Clinical Trial," JAMA Netw Open, vol. 1, no. 5, p. e181617, Sep 7 2018, doi: 10.1001/jamanetworkopen.2018.1617.

[36] B. M. Mehos, J. J. Saseen, and E. J. MacLaughlin, "Effect of pharmacist intervention and initiation of home blood pressure monitoring in patients with uncontrolled hypertension," Pharmacotherapy, vol. 20, no. 11, pp. 1384-9, Nov 2000, doi: 10.1592/phco.20.17.1384.34891.

[37] R. M. Peters et al., "Assessing the Utility of a Novel SMS- and Phone-Based System for Blood Pressure Control in Hypertensive Patients: Feasibility Study," JMIR Cardio, vol. 1, no. 2, p. e2, Jul 27 2017, doi: 10.2196/cardio.7915.

[38] J. D. Ralston et al., "Home blood pressure monitoring, secure electronic messaging and medication intensification for improving hypertension control: a mediation analysis," Appl Clin Inform, vol. 5, no. 1, pp. 232-48, 2014, doi: 10.4338/ACI-2013-10-RA-0079.

[39] K. Asayama et al., "Cardiovascular outcomes in the first trial of antihypertensive therapy guided by self-measured home blood pressure," Hypertens Res, vol. 35, no. 11, pp. 1102-10, Nov 2012, doi: 10.1038/hr.2012.125.

[40] K. Kario et al., "Digital therapeutics for essential hypertension using a smartphone application: A randomized, open-label, multicenter pilot study," J Clin Hypertens (Greenwich), vol. 23, no. 5, pp. 923-934, May 2021, doi: 10.1111/jch.14191.

[41] B. McKinstry et al., "Telemonitoring based service redesign for the management of uncontrolled hypertension: multicentre randomized controlled trial," BMJ, vol. 346, p. f3030, May 24 2013, doi: 10.1136/bmj.f3030.

[42] R. J. McManus et al., "Home and Online Management and Evaluation of Blood Pressure (HOME BP) using a digital intervention in poorly controlled hypertension: randomized controlled trial," BMJ, p. m4858, 2021, doi: 10.1136/bmj.m4858.

[43] R. J. McManus et al., "Efficacy of self-monitored blood pressure, with or without telemonitoring, for titration of antihypertensive medication (TASMINH4): an unmasked randomized controlled trial," Lancet, vol. 391, no. 10124, pp. 949-959, Mar 10 2018, doi: 10.1016/S0140-6736(18)30309-X.

[44] P. M. Moreira et al., "Optimizing Hypertension Treatment in Older Patients Through Home Blood Pressure Monitoring by Pharmacists in Primary Care: The MINOR Clinical Trial," Clin Ther, vol. 45, no. 10, pp. 941-946, Oct 2023, doi: 10.1016/j.clinthera.2023.06.007.

[45] C. L. Neumann et al., "Blood pressure telemonitoring is useful to achieve blood pressure control in inadequately treated patients with arterial hypertension," J Hum Hypertens, vol. 25, no. 12, pp. 732-8, Dec 2011, doi: 10.1038/jhh.2010.119.

[46] G. Parati et al., "Home blood pressure telemonitoring improves hypertension control in general practice. The TeleBPCare study," J Hypertens, vol. 27, no. 1, pp. 198-203, Jan 2009, doi: 10.1097/hjh.0b013e3283163caf.

[47] S. Rinfret et al., "The impact of a multidisciplinary information technology-supported program on blood pressure control in primary care," Circ Cardiovasc Qual Outcomes, vol. 2, no. 3, pp. 170-7, May 2009, doi: 10.1161/CIRCOUTCOMES.108.823765.

[48] S. W. Tobe, K. Hunter, R. Geerts, N. Raymond, and G. Pylypchuk, "IMPPACT: Investigation of Medical Professionals and Patients Achieving Control Together," Canadian Journal of Cardiology, vol. 24, no. 3, pp. 205-208, 2008, doi: 10.1016/s0828-282x(08)70585-3.

[49] J. F. Lu, C. M. Chen, and C. Y. Hsu, "Effect of home telehealth care on blood pressure control: A public healthcare centre model," J Telemed Telecare, vol. 25, no. 1, pp. 35-45, Jan 2019, doi: 10.1177/1357633X17734258.

[50] M. Wijkman, M. Carlsson, G. Darwiche, and F. H. Nystrom, "A pilot study of hypertension management using a telemedicine treatment approach," Blood Press Monit, vol. 25, no. 1, pp. 18-21, Feb 2020, doi: 10.1097/MBP.0000000000000413.

[51] M. Willis, G. Darwiche, M. Carlsson, A. Nilsson, J. Wohlin, and P. Lindgren, "Real-world long-term effects on blood pressure and other cardiovascular risk factors for patients in digital therapeutics," Blood Press Monit, vol. 28, no. 2, pp. 86-95, Apr 1 2023, doi: 10.1097/MBP.0000000000000633.

[52] V. H. Teo et al., "Effects of technology-enabled blood pressure monitoring in primary care: A quasi-experimental trial," J Telemed Telecare, vol. 30, no. 1, pp. 121-130, Jan 2024, doi: 10.1177/1357633X211031780.

[53] M. Xiao et al., "Home Blood Pressure Monitoring by a Mobile-Based Model in Chongqing, China: A Feasibility Study," Int J Environ Res Public Health, vol. 16, no. 18, Sep 10 2019, doi: 10.3390/ijerph16183325.

[54] A. Carrera et al., "BPcontrol. A Mobile App to Monitor Hypertensive Patients," Appl Clin Inform, vol. 7, no. 4, pp. 1120-1134, Dec 7 2016, doi: 10.4338/ACI-2015-12-RA-0172.
